# Definition of large bowel obstruction by primary colorectal cancer: A systematic review

**DOI:** 10.1111/codi.15479

**Published:** 2021-01-15

**Authors:** Joyce V. Veld, Kim J. Beek, Esther C.J. Consten, Frank ter Borg, Henderik L. van Westreenen, Wilhelmus A. Bemelman, Jeanin E. van Hooft, Pieter J. Tanis

**Affiliations:** ^1^ Department of Surgery Cancer Center Amsterdam Amsterdam UMC University of Amsterdam Amsterdam The Netherlands; ^2^ Department of Gastroenterology and Hepatology Cancer Center Amsterdam Amsterdam UMC University of Amsterdam Amsterdam The Netherlands; ^3^ Department of Gastroenterology and Hepatology NWZ Alkmaar Alkmaar The Netherlands; ^4^ Department of Surgery Meander Medical Center Amersfoort The Netherlands; ^5^ Department of Surgery University Medical Center Groningen Groningen The Netherlands; ^6^ Department of Gastroenterology and Hepatology Deventer Hospital Deventer The Netherlands; ^7^ Department of Surgery Isala Zwolle Zwolle The Netherlands; ^8^ Department of Gastroenterology and Hepatology Leiden University Medical Center Leiden The Netherlands

## Abstract

**Aim:**

Controversies on therapeutic strategy for large bowel obstruction by primary colorectal cancer mainly concern acute conditions, being essentially different from subacute obstruction. Clearly defining acute obstruction is important for design and interpretation of studies as well as for guidelines and daily practice. This systematic review aimed to evaluate definitions of obstruction by colorectal cancer in prospective studies.

**Method:**

A systematic search was performed in PubMed, Embase and the Cochrane Library. Eligibility criteria included randomized or prospective observational design, publication between 2000 and 2019, and the inclusion of patients with an obstruction caused by colorectal cancer. Provided definitions of obstruction were extracted with assessment of common elements.

**Results:**

A total of 16 randomized controlled trials (RCTs) and 99 prospective observational studies were included. Obstruction was specified as acute in 28 studies, complete/emergency in five, (sub)acute or similar terms in four and unspecified in 78. Five of 16 RCTs (31%) and 37 of 99 cohort studies (37%) provided a definition. The definitions included any combination of clinical symptoms, physical signs, endoscopic features and radiological imaging findings in 25 studies. The definition was only based on clinical symptoms in 11 and radiological imaging in six studies. Definitions included a radiological component in 100% of evaluable RCTs (5/5) vs. 54% of prospective observational studies (20/37, *P* = 0.07).

**Conclusion:**

In this systematic review, the majority of prospective studies did not define obstruction by colorectal cancer and its urgency, whereas provided definitions varied hugely. Radiological confirmation seems to be an essential component in defining acute obstruction.

## INTRODUCTION

Approximately 10% of patients with colorectal cancer present with acute large bowel obstruction. Different incidences have been reported [1], as the severity or degree of obstruction varies substantially and may significantly influence clinical decision‐making. Complete obstruction may lead to extensive bowel dilatation, even leading to caecal blow‐out, which increases the urgency of surgery or placement of a colonic self‐expandable metal stent (SEMS). In contrast, incomplete or imminent obstruction may allow for more conservative treatment including laxatives with subsequent surgery in a semi‐elective setting.

Clinical presentation of large bowel obstruction by primary colorectal cancer varies hugely in daily practice. Patients may present mainly with abdominal pain for several weeks, whereas others rapidly develop abdominal distention and might experience disproportionately mild symptoms. Conflicting results have been published regarding the degree of colorectal obstruction and SEMS success rates [2], which might be explained by variation in clinical presentation. Recently, a scoring system was developed by a Japanese group (the ColoRectal Obstruction Scoring System, CROSS) in order to evaluate the degree of colorectal obstruction and consequently aid in the choice of treatment of patients with obstructive colorectal cancer [3]. In their most recent guideline, the European Society of Gastrointestinal Endoscopy (ESGE) recommends against prophylactic SEMS placement in patients with a subacute obstruction [2], but without providing a clear definition.

A clear definition of obstruction by colorectal cancer might reduce overtreatment of patients with mild conditions, and focuses the need for emergency treatment towards patients with an acute presentation. In addition, literature on patients with obstructive colorectal cancer can be compared more effectively with more appropriate translation into clinical guidelines and daily practice. Furthermore, it enables clinical benchmarking. Therefore, the aim of this systematic review was to provide a literature overview of used definitions of obstruction by colorectal cancer in prospective studies, thereby serving as a basis for the development of a consensus definition.

## METHODS

This systematic review was reported according to the Preferred Reporting Items for Systematic Reviews and Meta‐Analyses (PRISMA) guidelines [4]. No review protocol was registered.

### Search strategy and data extraction

A systematic search was undertaken on MEDLINE/Pubmed, Embase and the Cochrane Library. The final search was performed on 13 March 2019. In MEDLINE/Pubmed, the following search terms were used: ((colon*[tiab] OR colorectal[tiab]) AND (cancer*[tiab] OR neoplasm*[tiab] OR malignan*[tiab]) AND obstructi*[tiab] AND english[language]). The first two authors (JV and KB) independently assessed titles and abstracts and subsequently full texts for eligibility. Reference lists of the included articles were manually cross‐searched for any additional studies. Any discrepancies were discussed and, in the case of disagreement, consensus was reached after consulting the senior author (PT).

### Inclusion/exclusion criteria

Studies were included in the case of a randomized or prospective observational study design, a publication date between 1 January 2000 and 13 March 2019, and if describing patients with any sign of obstruction caused by either right‐sided colon cancer, left‐sided colon cancer or rectal cancer. Exclusion criteria were retrospective studies, reviews, letters, editorials, studies including patients <18 years old, animal studies and studies not written in English. Not providing a definition of obstructive colon cancer was not an exclusion criterion.

### Outcomes of interest

Provided definitions of malignant colorectal obstruction or any related descriptions of the clinical condition and its urgency of included patients were extracted from each study. Definitions were evaluated based on whether or not they contained any of the following five elements: clinical symptoms, duration of symptoms, physical examination, endoscopic features and/or radiological imaging. Other extracted variables included study design, number of patients, terminology used for the studied clinical condition as mentioned in the Methods section, and whether or not a definition of obstructive colon cancer was provided in the Methods section.

## RESULTS

### Study selection

**FIGURE 1:**
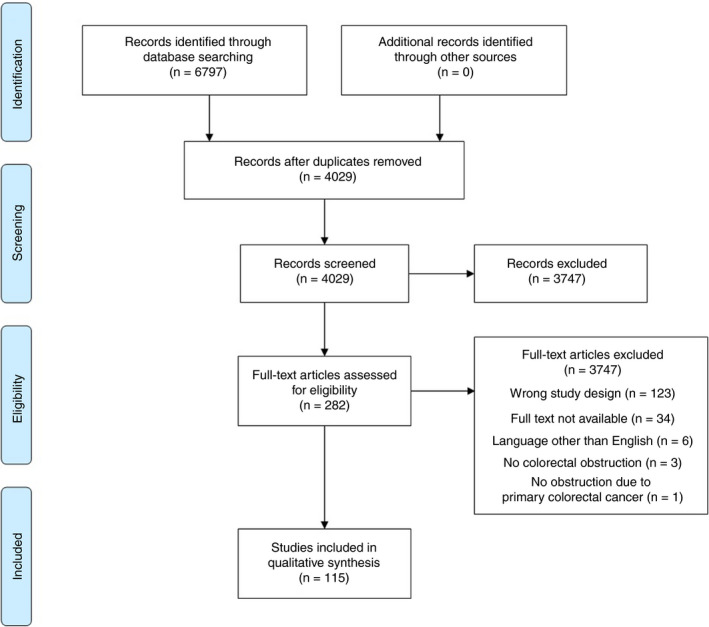
PRISMA flowchart

The literature search yielded a total of 6797 articles (Figure 1). After exclusion of 2768 duplicates, 4029 articles were screened on title and abstract, and 282 studies remained for full text screening. After exclusion based on study design (*n* = 123), 115 studies remained for final analysis including 16 randomized controlled trials (RCTs) (13.9%) and 99 prospective observational studies (86.1%). The studies are summarized in Table 1.

**TABLE 1:** Study characteristics of all the included studies

Author	Year	Title
Bayrak	2019	Stent experiences in emergency conditions in acute mechanical intestinal obstruction caused by colorectal cancer.
Fiori	2018	Endoscopic Stenting for Colorectal Cancer
Takahashi	2018	Oncological Assessment of Stent Placement for Obstructive Colorectal Cancer from Circulating Cell‐Free DNA and Circulating Tumor DNA Dynamics.
Yamashita	2018	Impact of endoscopic stent insertion on detection of viable circulating tumor cells from obstructive colorectal cancer.
Li	2017	Clinical application of transanal ileal tube placement using X‐ray monitoring.
Arezzo	2016	Colonic stenting as a bridge to surgery versus emergency surgery for malignant colonic obstruction: results of a multicentre randomised controlled trial (ESCO trial)
Enomoto	2016	Open surgery versus laparoscopic surgery after stent insertion for obstructive colorectal cancer.
Haraguchi	2016	Colonic stenting as a bridge to surgery for obstructive colorectal cancer: advantages and disadvantages.
Saito	2016	A prospective multicenter study on self‐expandable metallic stents as a bridge to surgery for malignant colorectal obstruction in Japan: efficacy and safety in 312 patients.
Wan	2016	Comparison of through‐the‐scope stent insertion with standard stent insertion for the management of malignant colorectal obstruction: a prospective study.
Achiam	2015	Perioperative Colonic Evaluation in Patients with Rectal Cancer; MR Colonography Versus Standard Care.
Lin	2015	Investigation of treatment methods in obstructive colorectal cancer.
Matsuzawa	2015	A Japanese prospective multicenter study of self‐expandable metal stent placement for malignant colorectal obstruction: short‐term safety and efficacy within 7 days of stent procedure in 513 cases.
Milek	2015	Implantation of a new enteral stent in obstructive colorectal cancer using interventional radiology in patients over 70 years of age.
Singh	2015	Role of CT Colonography in Colonic Lesions and Its Correlation with Conventional Colonoscopic Findings.
Young	2015	Improving Quality of Life for People with Incurable Large‐Bowel Obstruction: Randomized Control Trial of Colonic Stent Insertion.
Di Mitri	2014	The new nitinol conformable self‐expandable metal stents for malignant colonic obstruction: a pilot experience as bridge to surgery treatment.
Kamocki	2014	Own experiences of endoscopic self‐expandable stent placement for malignant colorectal ileus.
Kim	2014	Mmp‐9 expression after metallic stent placement in patients with colorectal cancer: association with in‐stent restenosis.
Kim	2014	Preoperative colonoscopy through the colonic stent in patients with colorectal cancer obstruction.
Krstic	2014	Hartmann's procedure vs loop colostomy in the treatment of obstructive rectosigmoid cancer.
Lo	2014	Protocol‐driven self‐expanding metallic stenting for malignant large‐bowel obstruction in a district hospital
Occhionorelli	2014	Colonic stent placement as a bridge to surgery in patients with left‐sided malignant large bowel obstruction. An observational study.
Ding	2013	A temporary self‐expanding metallic stent for malignant colorectal obstruction.
Ghazal	2013	Colonic endolumenal stenting devices and elective surgery versus emergency subtotal/total colectomy in the management of malignant obstructed left colon carcinoma.
Lamazza	2013	A new technique for placement of a self‐expanding metallic stent (SEMS) in patients with colon rectal obstruction: a prospective study of 43 patients.
Lee	2013	Novel method of stent insertion for malignant lower rectal obstruction with proximal releasing delivery system (with video).
Lim	2013	Preoperative colonoscopy for detection of synchronous neoplasms after insertion of self‐expandable metal stents in occlusive colorectal cancer: comparison of covered and uncovered stents.
Sumise	2013	Outcome of emergency one‐stage resection and anastomosis procedure for patients with obstructed colorectal cancer.
Warden	2013	Stenting as first‐line management for all patients with nonperforating left‐sided obstructing colorectal cancer.
Yoshida	2013	Feasibility of a new self‐expandable metallic stent for patients with malignant colorectal obstruction.
Angenete	2012	Stenting for colorectal cancer obstruction compared to surgery‐‐a study of consecutive patients in a single institution.
Bonfante	2012	Managing acute colorectal obstruction by "bridge stenting" to laparoscopic surgery: Our experience
Cennamo	2012	Colorectal stenting as a bridge to surgery reduces morbidity and mortality in left‐sided malignant obstruction: a predictive risk score‐based comparative study.
Chen	2012	Laparoscopic management for acute malignant colonic obstruction.
Cheung	2012	Outcome and safety of self‐expandable metallic stents for malignant colon obstruction: a Korean multicenter randomized prospective study.
Chou	2012	Dual‐design expandable colorectal stent for a malignant colorectal obstruction: preliminary prospective study using new 20‐mm diameter stents.
Ho	2012	Endoscopic stenting and elective surgery versus emergency surgery for left‐sided malignant colonic obstruction: a prospective randomized trial.
Inaba	2012	Phase II clinical study on stent therapy for unresectable malignant colorectal obstruction (JIVROSG‐0206).
JimenezFuertes	2012	Resection and primary anastomosis without diverting ileostomy for left colon emergencies: is it a safe procedure?
Larssen	2012	Long‐term outcome of palliative treatment with self‐expanding metal stents for malignant obstructions of the GI tract.
Meisner	2012	Self‐Expanding Metal Stenting for Palliation of Patients with Malignant Colonic Obstruction: Effectiveness and Efficacy on 255 Patients with 12‐Month's Follow‐up.
Song	2012	Usefulness of a guiding sheath for fluoroscopic colorectal stent placement.
Tominaga	2012	Favorable long‐term clinical outcome of uncovered D‐weave stent placement as definitive palliative treatment for malignant colorectal obstruction.
Yang	2012	Two‐stage resection for malignant colonic obstructions: the timing of early resection and possible predictive factors.
Alcantara	2011	Prospective, controlled, randomized study of intraoperative colonic lavage versus stent placement in obstructive left‐sided colonic cancer.
Chakraborty	2011	Malignant bowel obstruction: natural history of a heterogeneous patient population followed prospectively over two years.
Luigiano	2011	Through‐the‐scope large diameter self‐expanding metal stent placement as a safe and effective technique for palliation of malignant colorectal obstruction: a single center experience with a long‐term follow‐up.
Meisner	2011	Self‐expandable metal stents for relieving malignant colorectal obstruction: short‐term safety and efficacy within 30 days of stent procedure in 447 patients.
Milek	2011	Preliminary results of the use of self‐expanding nitinol stents in inoperable gastrointestinal cancers
Pirlet	2011	Emergency preoperative stenting versus surgery for acute left‐sided malignant colonic obstruction: a multicenter randomized controlled trial.
Sule	2011	Adult large bowel obstruction: a review of clinical experience.
van Hooft	2011	Colonic stenting versus emergency surgery for acute left‐sided malignant colonic obstruction: a multicentre randomised trial.
Williams	2011	Colorectal stenting in malignant large bowel obstruction: the learning curve.
Young	2011	Stenting large bowel obstruction avoids a stoma: consecutive series of 100 patients.
Achiam	2010	Differentiation between benign and malignant colon tumors using fast dynamic gadolinium‐enhanced MR colonography; a feasibility study.
Branger	2010	Management of acute malignant large‐bowel obstruction with self‐expanding metal stent.
Dakubo	2010	Colorectal carcinoma: an update of current trends in Accra.
Hisanaga	2010	Multicenter prospective study on efficacy and safety of octreotide for inoperable malignant bowel obstruction.
Li	2010	Management of acute malignant colorectal obstruction with a novel self‐expanding metallic stent as a bridge to surgery.
Moon	2010	Comparison of a newly designed double‐layered combination covered stent and D‐weave uncovered stent for decompression of obstructive colorectal cancer: a prospective multicenter study
Mukai	2010	Two‐stage treatment (Mukai's method) with hybrid 2‐port HALS (Mukai's operation) for complete bowel obstruction by left colon cancer or rectal cancer.
Nagula	2010	Quality of life and symptom control after stent placement or surgical palliation of malignant colorectal obstruction.
Park	2010	Comparison of efficacies between stents for malignant colorectal obstruction: a randomized, prospective study.
Tanaka	2010	Endoscopic balloon dilation for obstructive colorectal cancer: a basic study on morphologic and pathologic features associated with perforation.
Brehant	2009	Elective (planned) colectomy in patients with colorectal obstruction after placement of a self‐expanding metallic stent as a bridge to surgery: the results of a prospective study.
Cheung	2009	Endolaparoscopic approach vs conventional open surgery in the treatment of obstructing left‐sided colon cancer: a randomized controlled trial.
Kim	2009	Dual‐design expandable colorectal stent for malignant colorectal obstruction: comparison of flared ends and bent ends.
Maras‐Simunic	2009	Use of modified multidetector CT colonography for the evaluation of acute and subacute colon obstruction caused by colorectal cancer: a feasibility study.
Marelli	2009	Clinical utility of serum tumor markers in the diagnosis of malignant intestinal occlusion. A prospective observational study.
Reza	2009	Colorectal stenting for management of acute malignant bowel obstruction in advanced colorectal cancer in Iran.
Stenhouse	2009	Self expanding wall stents in malignant colorectal cancer: is complete obstruction a contraindication to stent placement?
Baraza	2008	Combination endo‐radiological colorectal stenting: a prospective 5‐year clinical evaluation.
Elsberger	2008	Self‐expanding metallic stent insertion in the proximal colon
Fregonese	2008	Ultraflex precision colonic stent placement as a bridge to surgery in patients with malignant colon obstruction.
Im	2008	Clinical outcomes and patency of self‐expanding metal stents in patients with malignant colorectal obstruction: a prospective single center study.
Jiang	2008	Primary vs. delayed resection for obstructive left‐sided colorectal cancer: impact of surgery on patient outcome.
Nagata	2008	PET/CT colonography for the preoperative evaluation of the colon proximal to the obstructive colorectal cancer.
Repici	2008	WallFlex colonic stent placement for management of malignant colonic obstruction: a prospective study at two centers.
Stipa	2008	Management of obstructive colorectal cancer with endoscopic stenting followed by single‐stage surgery: open or laparoscopic resection?
van Hooft	2008	Early closure of a multicenter randomized clinical trial of endoscopic stenting versus surgery for stage IV left‐sided colorectal cancer.
Varpe	2008	Adoption of self‐expanding metallic stents in the palliative treatment of obstructive colorectal cancerg‐look out for perforations!
Wong	2008	Tumor pathology and long‐term survival in emergency colorectal cancer
Alcantara	2007	Colorectal stenting as an effective therapy for preoperative and palliative treatment of large bowel obstruction: 9 years' experience.
Choi	2007	Interventional management of malignant colorectal obstruction: use of covered and uncovered stents.
Lee	2007	Comparison of uncovered stent with covered stent for treatment of malignant colorectal obstruction.
Mitchell	2007	Emergency room presentation of colorectal cancer: a consecutive cohort study.
Mucci‐Hennekinne	2007	Management of acute malignant large‐bowel obstruction with self‐expanding metal stent.
Olmi	2007	Acute colonic obstruction: endoscopic stenting and laparoscopic resection.
Repici	2007	Ultraflex precision colonic stent placement for palliation of malignant colonic obstruction: a prospective multicenter study.
Song	2007	A dual‐design expandable colorectal stent for malignant colorectal obstruction: results of a multicenter study.
Tsurumaru	2007	Self‐expandable metallic stents as palliative treatment for malignant colorectal obstruction.
McArdle	2006	The impact of blood loss, obstruction and perforation on survival in patients undergoing curative resection for colon cancer.
Ptok	2006	Palliative stent implantation in the treatment of malignant colorectal obstruction.
Vitale	2006	Preoperative colonoscopy after self‐expandable metallic stent placement in patients with acute neoplastic colon obstruction.
Davies	2005	Bowel function following insertion of self‐expanding metallic stents for palliation of colorectal cancer.
Gallardo‐Valverde	2005	Obstruction in patients with colorectal cancer increases morbidity and mortality in association with altered nutritional status.
Kim	2005	Complete single‐stage management of left colon cancer obstruction with a new device.
Lim	2005	Prospective, randomized trial comparing intraoperative colonic irrigation with manual decompression only for obstructed left‐sided colorectal cancer.
Poon	2005	Evaluation of P‐POSSUM in surgery for obstructing colorectal cancer and correlation of the predicted mortality with different surgical options.
Syn	2005	Metallic stents in large bowel obstruction: Experience in a District General Hospital
Villar	2005	Surgical options for malignant left‐sided colonic obstruction.
Balague	2004	Minimally invasive treatment for obstructive tumors of the left colon: endoluminal self‐expanding metal stent and laparoscopic colectomy. Preliminary results.
Law	2004	Palliation for advanced malignant colorectal obstruction by self‐expanding metallic stents: prospective evaluation of outcomes.
Maeda	2004	Successful treatment using a self‐expandable metallic stent in the palliation for unresectable malignant obstruction of the colon and rectum
Meisner	2004	Self‐expanding metal stents for colonic obstruction: experiences from 104 procedures in a single center.
Park	2004	Single‐stage procedure with intraoperative colonoscopy and colonic irrigation in patients with obstructing left‐sided colonic cancer.
Shim	2004	Through‐the‐scope double colonic stenting in the management of inoperable proximal malignant colonic obstruction: a pilot study.
Tekkis	2004	The Association of Coloproctology of Great Britain and Ireland study of large bowel obstruction caused by colorectal cancer.
Tomiki	2004	Comparison of stent placement and colostomy as palliative treatment for inoperable malignant colorectal obstruction.
Vanbiervliet	2004	Endoscopic palliative treatment of malignant colorectal stenosis with metallic stents: Results in 41 patients
Xinopoulos	2004	Stenting or stoma creation for patients with inoperable malignant colonic obstructions? Results of a study and cost‐effectiveness analysis.
Law	2003	Comparison of stenting with emergency surgery as palliative treatment for obstructing primary left‐sided colorectal cancer.
DeGregorio	2002	Use of an introducer sheath for colonic stent placement.
Martinez‐Santos	2002	Self‐expandable stent before elective surgery vs. emergency surgery for the treatment of malignant colorectal obstructions: comparison of primary anastomosis and morbidity rates.
Seymour	2002	Palliative stenting of malignant large bowel obstruction
Vrazas	2002	Stenting for obstructing colorectal malignancy: an interim or definitive procedure.
Wong	2002	Treatment of acute malignant colorectal obstruction with self‐expandable metallic stents.
Carraro	2001	Obstructing colonic cancer: failure and survival patterns over a ten‐year follow‐up after one‐stage curative surgery.
Mao	2001	Treatment of malignant digestive tract obstruction by combined intraluminal stent installation and intra‐arterial drug infusion.
Tanaka	2001	Endoscopic transanal decompression with a drainage tube for acute colonic obstruction: Clinical aspects of preoperative treatment
Camunez	2000	Malignant colorectal obstruction treated by means of self‐expanding metallic stents: effectiveness before surgery and in palliation.
Grunshaw	2000	Prospective evaluation of ultrasound in distal ileal and colonic obstruction
Laval	2000	The use of steroids in the management of inoperable intestinal obstruction in terminal cancer patients: do they remove the obstruction?
Law	2000	Self‐expanding metallic stent in the treatment of colonic obstruction caused by advanced malignancies.
Repici	2000	Covered metal stents for management of inoperable malignant colorectal strictures.
Tamim	2000	Experience with endoluminal colonic wall stents for the management of large bowel obstruction for benign and malignant disease.

### Terminology

Main terms for the clinical condition that were adhered to in the included studies were ‘obstruction/obstructing/obstructive’ (*n* = 105), ‘stricture’ (*n* = 5) and ‘occlusion’ (*n* = 2) (Table 1). ‘Ileus’ was not used in any of the included studies. In five studies, no specific terminology was provided in the Methods section. The urgency of the clinical condition was further specified in 34 studies: ‘acute’ (*n* = 28), ‘complete’ (*n* = 4), ‘emergency’ (*n* = 1), ‘imminent’ (*n* = 1). Three studies included patients of varying urgency (‘(sub)acute’, ‘(in)complete’, ‘(sub)occlusion’).

### Definition of obstructive colorectal cancer

**FIGURE 2:**
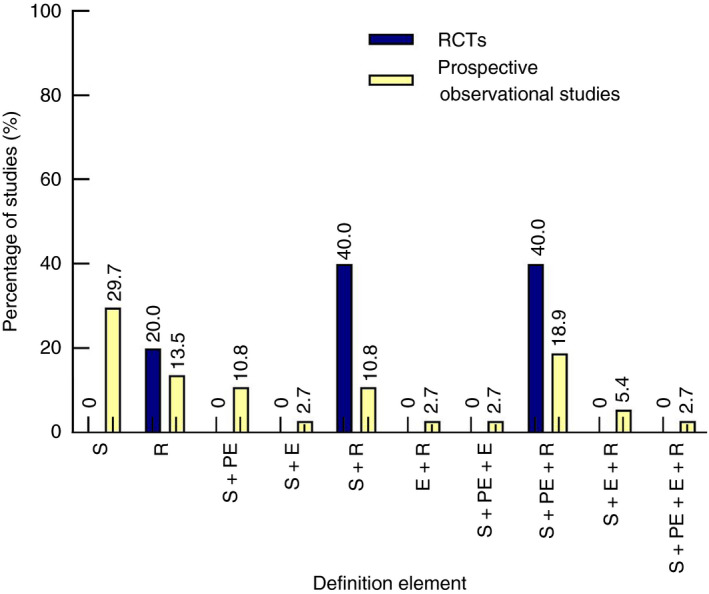
Percentage of studies reporting each (combination of) element(s) within their definition

In total, 42 of the 115 included studies (36.5%) provided a definition of obstructive colorectal cancer: five of 16 RCTs (31.3%) and 37 of 99 prospective observational studies (37.4%) (Table 1). The definitions of these 42 studies are displayed in Table 2, thereby separating the used terms and descriptions for each of the five predefined elements. The definition was solely based on clinical symptoms in 11 studies [5, 6, 7, 8, 9, 10, 11, 12, 13, 14, 15] or solely on radiological imaging in six studies [16, 17, 18, 19, 20, 21] (Figure 2). In the remaining studies, a combination of clinical symptoms with or without duration, physical examination, endoscopic features or radiological imaging was incorporated into the definition of obstruction. A combination of symptoms and physical examination was used in four studies [22, 23, 24, 25], symptoms including duration and physical examination in two studies [23, 25], symptoms and endoscopic features in one study [26], symptoms including duration and endoscopic features in none of the studies, symptoms and radiological findings in six studies [27, 28, 29, 30, 31, 32], symptoms including duration and radiological findings in two studies [28, 29], symptoms, physical examination and endoscopic features in one study [33], and symptoms, physical examination and radiological findings in nine studies [34, 35, 36, 37, 38, 39, 40, 41, 42], with three of these also including duration of symptoms [34, 38, 41]. None of the studies included all five elements. A radiological component was incorporated in the used definitions in 100% of RCTs (5/5) vs. 54.1% of prospective observational studies (20/37, *P* = 0.07). No studies were identified that formally validated a definition of obstructive colorectal cancer.

**TABLE 2:** Definitions provided for colorectal obstruction in prospective studies, divided into five predefined elements

	First author	Year	Clinical symptoms	Duration of symptoms	Physical examination	Endoscopic features	Radiological imaging
1	Bayrak [34]	2019	No bowel movements during last 24 h, fecaloid vomiting	During last 24 h	Abdominal distention	NR	Enlarged colonic loops
2	Fiori [8]	2018	Symptoms of severe obstruction lasting for >3 months	>3 months	NR	NR	NR
3	Takahashi [45]	2018	Pain	NR	Abdominal distention	Severe colonic obstruction on endoscopic imaging	Dilatation of the colon on plain or enhanced abdominal CT scan
4	Saito [12]	2016	CROSS	NR	NR	NR	NR
5	Matsuzawa [43]	2015	Complete obstruction: inability to pass flatus [50] Remaining cases: incomplete obstruction + CROSS	NR	NR	Complete obstruction: inability to endoscopically visualize the proximal lumen [50] Remaining cases: incomplete obstruction	Complete obstruction: inability of water‐soluble contrast to pass proximal to the lesion [50] Remaining cases: incomplete obstruction
6	Di Mitri [6]	2014	Unable to pass stool and gas, vomiting, abdominal pain, paradoxical diarrhoea	NR	NR	NR	NR
7	Kim [33]	2014	Constipation	NR	Abdominal distention	Endoscopic features of colonic obstruction and colonoscope with diameter 12.2 or 13.2 mm could not pass through the stricture	NR
8	Ding [7]	2013	Symptoms resulting in difficulty in defaecation	NR	NR	NR	NR
9	Yoshida [44]	2013	Complete obstruction: inability to pass flatus Remaining cases: incomplete obstruction [50]	NR	NR	Complete obstruction: lack of endoscopically visible proximal lumen Remaining cases: incomplete obstruction [50]	Complete obstruction: lack of water‐soluble contrast passing proximally to the lesion Remaining cases: incomplete obstruction [50]
10	Cennamo [35]	2012	Abdominal pain, complete blockage of bowel transit and flatus	NR	Abdominal distention	NR	Dilated bowel proximal to the site of obstruction
11	Chou [5]	2012	Symptoms resulting in defaecation difficulty	NR	NR	NR	NR
12	Ho [37]	2012	Vomiting, abdominal pain, inability to pass stools	NR	Abdominal distention	NR	Dilated colon
13	Song [14]	2012	Symptoms that resulted in defaecation difficulty	NR	NR	NR	NR
14	Alcantara [21]	2011	NR	NR	NR	NR	Diagnosis of complete intestinal obstruction due to tumour in left colon using abdominal CT [48]
15	Chakraborty [36]	2011	Nausea, vomiting, pain [49]	NR	Abdominal distention [49]	NR	Corresponding findings on CT scan of abdomen and pelvis [49]
16	Marrelli [39]	2011	Abdominal pain, constipation	NR	Abdominal distention	NR	Air–fluid levels suggestive of mechanical ileus
17	Sule [42]	2011	Constipation, abdominal pain, nausea	NR	Abdominal distention	NR	Radiographic features of large bowel obstruction
18	Van Hooft [29]	2011	Clinical signs of severe colonic obstruction that had existed for less than 1 week	<1 week	NR	NR	Dilatation of the colon on either plain abdominal radiograph, with typical abnormalities on a gastrografin enema study, or contrast‐enhanced CT scan
19	Hisanaga [22]	2010	Nausea/vomiting or abdominal pain	NR	Abdominal distention	NR	NR
20	Nagula [24]	2010	Progressive constipation, multiple small bowel movements daily, abdominal pain, and nausea and vomiting	NR	Abdominal distention	NR	NR
21	Brehant [17]	2009	NR	NR	NR	NR	Colon distention, upstream malignant stenosis
22	Kim [10]	2009	Symptoms resulting in difficulty in defaecation	NR	NR	NR	NR
23	Maras‐Simunic [15]	2009	Altered bowel habits	NR	NR	NR	NR
24	Stenhouse [19]	2009	NR	NR	NR	NR	Complete obstruction: no retrograde flow of gastrografin or bowel dilatation proximal to a transition zone followed by distal bowel collapse on CT Incomplete obstruction: partially obstructing lesion with retrograde flow of gastrografin
25	Fregonese [9]	2008	Abdominal/perianal pain, nausea, vomiting, bloating, decreased stool calibre, haematochezia, tenesmus, urgency, rectal bleeding, incontinence, melena	NR	NR	NR	NR
26	Nagata [18]	2008	NR	NR	NR	NR	Bowel distention in segments proximal vs. distal from the lesion
27	Repici [41]	2008	Inability to pass flatus or stool through the anus for more than 24 h	> 24 h	Bowel distention	NR	Radiographic evidence of ileus
28	Alcantara [16]	2007	NR	NR	NR	NR	Only provided for incomplete obstruction: bowel dilatation seen at CT with some contrast medium passing through the tumour
29	Lee [23]	2007	Obstipation or constipation >48 h, nausea, vomiting or cramping abdominal pain	> 48 h	Abdominal distention	NR	NR
30	Olmi [25]	2007	Symptoms such as severe constipation longer than 48 h, nausea, vomiting and cramping abdominal pain	> 48 h	Abdominal distention	NR	NR
31	Song [13]	2007	Difficulty in defaecation	NR	NR	NR	NR
32	Ptok [46]	2006	NR	NR	NR	Tumour stenosis that could not be passed with a normal colonoscope	Manifest ileus or prestenotic dilatation of the colon exceeding 6 cm
33	Kim [26]	2005	Complete obstruction: NR Nearly complete obstruction: abdominal pain in bowel preparation for colon study	NR	NR	Complete obstruction: NR Nearly complete obstruction: complete colonoscopic examination was impossible because of narrowing	NR
34	Lim [27]	2005	Delay of stool	NR	NR	NR	Large bowel distention on plain supine abdominal radiograph with confirmation on flexible sigmoidoscopy and/or CT of the abdomen or pelvis
35	Poon [40]	2005	Abdominal pain and constipation	NR	Abdominal distention	NR	Dilated bowel proximal to the site of obstruction
36	Shim [28]	2004	Constipation lasting longer than 48 h, vomiting or cramping abdominal pain	>48 h	NR	NR	Conventional radiographic evidence of colonic obstruction on a barium enema examination
37	Tekkis [32]	2004	Clinical or radiological evidence of distended large and/or small bowel secondary to colorectal cancer and presenting as an emergency	NR	NR	NR	Clinical or radiological evidence of distended large and/or small bowel secondary to colorectal cancer and presenting as an emergency
38	Tanaka [20]	2001	NR	NR	NR	NR	Bowel dilatation, formation of air–fluid level and presence of tumour with plain abdominal radiography and CT rather than contrast medium
39	Grunshaw [30]	2000	Clinical or radiological findings suggestive of distal small bowel or colonic obstruction	NR	NR	NR	Fluid or gas‐filled colon, terminating abruptly with collapsed colon distally, with or without the presence of a mass lesion
40	Laval [38]	2000	At least 3 criteria: vomiting at least twice a day, colicky abdominal pain, no flatus for 12 h or more, no stool for at least 4 days (excluding cases of faecal impaction)	At least 3 criteria: vomiting at least twice a day, colicky abdominal pain, no flatus for 12 h or more, no stool for at least 4 days (excluding cases of faecal impaction)	Intestinal distention	NR	Air–fluid levels or the absence of large bowel gas on abdominal X‐ray
41	Repici [11]	2000	Complete obstruction: no passage of gas and faeces Incomplete obstruction: only passage of gas	NR	NR	NR	NR
42	Tamim [31]	2000	Not passing stool or gas via the rectum	NR	NR	NR	Signs of complete obstruction on plain radiography, CT scan or contrast‐enhanced enema

CROSS, ColoRectal Obstruction Scoring System; NR, not reported.

### Clinical symptoms

The most frequently reported clinical symptoms included abdominal pain, inability to pass stool or flatus, nausea and/or vomiting. The CROSS score, which focuses on the level of oral intake along with symptoms of stricture, was used in two of 42 studies (4.8%) [12, 43]. In three of the studies [11, 43, 44], absence of flatus determined the degree of obstruction: if the patient was still able to pass flatus, the obstruction was incomplete. The required duration of symptoms for patients to be eligible for inclusion was reported in eight studies [8, 23, 25, 28, 29, 34, 38, 41]. Three studies mentioned >48 h as a minimum [23, 25, 28], whereas others used criteria such as symptoms for at least 12 h [38] or 24 h [34, 41], less than 1 week [29] or at least 3 months [8].

### Physical examination

Findings during physical examination that were included in definitions of colorectal obstruction included abdominal distention in 13 studies [22, 23, 24, 25, 39, 40, 42, 45], bowel distention in one study [41] and intestinal distention in one study [38].

### Endoscopic features

Endoscopic features were included in the definition of obstruction in six studies and were described as ‘the inability of the endoscopist to visualize the proximal lumen’ [43, 44], ‘a stenosis that could not be passed with a colonoscope’ [33, 46] or ‘severe colonic obstruction on endoscopic imaging’ [45]. One study specifically defined nearly complete obstruction using endoscopic features: ‘complete colonoscopic examination was impossible because of narrowing’ [26].

### Radiological imaging

Descriptions of findings during radiological imaging as one of the elements of the definition of obstruction included ‘enlarged colonic loops’, ‘dilated colon proximally to the lesion’, ‘termination of a fluid‐ or gas‐filled colon by a collapsed colon distally’, ‘inability of contrast to pass proximal to the lesion’, ‘some contrast passing the tumour in case of incomplete obstruction’ and ‘air–fluid levels suggestive of ileus’.

## DISCUSSION

The current systematic review reveals an important shortcoming in the available literature on large bowel obstruction by colorectal cancer regarding the definition of the relevant clinical condition, as well as the specification of the urgency of obstruction. Even after eligibility was confined to only randomized and prospective observational studies, we found that only 42 of the 115 included studies (36.5%) provided any definition of obstruction and only 34 studies (29.6%) specified the urgency. The proportion of studies providing any definition was similar for RCTs (31%) and non‐randomized observational cohort studies (37%). Definitions varied extensively regarding the terms and descriptions that were used, as well as the number and combinations of the five predefined elements that were incorporated. Most studies used a combination of clinical symptoms with or without duration and findings during radiological imaging to define malignant colorectal obstruction. All RCTs using a definition for obstruction incorporated radiological signs to confirm the presence of obstruction, while this was the case in only half of prospective observational studies. The required duration of symptoms for inclusion varied from 12 h up to 3 months.

Regarding the terminology of the clinical condition, ‘obstruction’ or similar words were mostly used. However, given the fact that this term can apparently be used independent of the degree of obstruction, it seems necessary to further specify this condition. Considering the desired relevance of the definition for therapeutic decision‐making, one should name the condition that indicates the necessity for an emergency intervention, either surgical or endoscopic. ‘Acute obstruction’ probably best expresses this clinical scenario, while ‘complete obstruction’ wrongly implies that one can reliably assess the degree of obstruction by any measure. If urgency was specified, ‘acute’ was used in the vast majority of studies.

Clinical symptoms that were included in the definitions were abdominal pain, nausea, vomiting and several words and phrases referring to problems with passing stools or flatus. Reporting these symptoms may vary between physicians according to differences in education, specialization and experience, besides geographical and psychosocial differences in the way patients present their symptoms, and thus be subject to inter‐observer variability. Some of the symptoms are relatively unspecific, such as abdominal pain and nausea. Vomiting is a more objective symptom, but only presents in the case of malignant obstruction if the small bowel dilates with insufficiency of the ileocaecal valve. Inability to pass stools or flatus might also be difficult to judge, and terms such as ‘constipation’, ‘change in bowel habits’ or ‘difficulty in defaecation’ do not seem to be appropriate to define acute obstruction.

Duration of symptoms was infrequently described and varied between 12 h and 3 months. Notably, several studies used a minimum of 24 h or even 12 h of not passing stools or flatus as a criterion for inclusion. However, this might also be the case in physiological circumstances, raising the question whether adding an interval of 24 h or less to the definition of colorectal obstruction regarding bowel movements is of any relevance. If one would consider including passage of stools or flatus as part of the definition of acute obstruction, the minimal duration should probably be at least 48 h.

Radiological confirmation of obstruction is probably one of the essential elements of the definition. However, in order to define the condition as an emergency, it also seems essential that symptoms should be included in a definition of acute obstruction in combination with at least the presence of abdominal distention during physical examination. The other way around, abdominal distention during physical examination in the absence of any related symptoms can contribute to defining acute colorectal obstruction.

In the case of emergency surgery, colonoscopy is often not performed before resection or construction of a decompressing stoma. This was confirmed by the current systematic review, in which endoscopic characteristics were infrequently part of the requirements for having obstructive colorectal cancer. Furthermore, endoscopic features such as inability to pass the endoscope might be observed in the absence of any clinical or radiological signs of obstruction. For these reasons, endoscopic features should probably not be included in the definition of acute obstruction. In contrast, a CT scan showing dilated colon proximal to a malignant appearing stenosis is probably one of the most reliable elements of a definition of obstruction [47]. The only difficulty for radiologists is to distinguish obstruction from colonic dilatation or pseudo‐obstruction, and a contrast enema can help to differentiate. Furthermore, the clinical diagnosis only fits into ‘acute obstruction’ in combination with at least one clinical criterion such as vomiting, distended abdomen or not passing stools or flatus for at least 48 h.

The required maximum interval between presentation and first intervention, reflecting urgency and severity of the obstruction, was reported even less frequently than duration of symptoms. In one RCT [29], patients had to be treated with either an SEMS or surgery within 24 h of randomization. Although such a criterion might add to the definition of acute obstruction, incorporating timing of subsequent therapeutic interventions is probably not the purest way of defining a clinical condition. Considering therapeutic consequences, it is also of relevance that the ESGE guideline of 2020 recommends against prophylactic colonic SEMS placement. According to the guideline, SEMS is only indicated in patients with both obstructive symptoms and radiological findings suspicious of malignant large bowel obstruction, because of the potential risks associated with colonic stenting [2]. An unclear distinction between acute and imminent obstruction may result in overtreatment of patients who might experience relief of mild symptoms by, for example, laxatives with subsequent semi‐elective surgery. This underlines the need to talk about ‘acute obstruction’, as mentioned before.

Recently, a scoring system CROSS was developed by a Japanese group in order to evaluate the degree of colorectal obstruction and consequently aid in the choice of treatment of patients with obstructive colorectal cancer [3]. This scoring system focuses on the level of oral intake along with symptoms of stricture, including abdominal pain or cramps, abdominal distention, nausea, vomiting, constipation and diarrhoea. The less able a patient is to eat soft solids, the lower the CROSS score. However, this scoring system is infrequently used in the literature. This was confirmed by the current systematic review, with only two of 42 studies (4.8%) adhering to the CROSS scoring system. A disadvantage of this scoring system is the lack of any radiological criteria. Before widespread implementation, such a scoring system has to be assessed regarding its relevance to therapeutic decision‐making and subsequent clinical outcome parameters. Subsequently, validation is required in different clinical settings.

The limitations of the present review are related to the selection of studies. Besides RCTs, we decided to include studies with a prospective study design. However, the term ‘prospective’ does not necessarily mean that a complete research protocol had been written before data collection and might only indicate that patients were prospectively identified. This might explain the low proportion of definitions provided, although the proportion was similar for RCTs. In addition, because of the rather long inclusion period, the methodological criteria of designing and reporting prospective studies as well as the diagnostic work‐up of such patients have probably changed over time. Furthermore, insight into the clinical relevance of the urgency of the obstruction might have increased.

In conclusion, obstruction by colorectal cancer was not clearly defined and its urgency was not specified in the majority of the included prospective studies in the current systematic review. Radiological imaging showing distended bowel proximal to a suspicious malignant stenosis seems an essential element of a definition of obstruction by colorectal cancer. If combined with a distended abdomen during physical examination with or without specific clinical symptoms, this can define the clinical condition of ‘acute obstruction’ with required relevance for therapeutic decision‐making. Consensus on one uniform definition is warranted, in order to reduce overtreatment of imminent obstruction, to improve comparability of the literature, to facilitate guideline development and to enable benchmarking within a clinical audit.

## CONFLICT OF INTERESTS

J.V. Veld, K.J. Beek, E.C.J. Consten, H.L. van Westreenen, F. ter Borg, W.A. Bemelman, J.E. van Hooft and P.J. Tanis have no conflicts of interests or financial ties to disclose for this specific study. Outside of the submitted work, J.E. van Hooft received a grant from Cook Medicals and a consultancy fee from Boston Scientific and Medtronics.

## ETHICAL APPROVAL

J.V. Veld, K.J. Beek, E.C.J. Consten, H.L. van Westreenen, F. ter Borg, W.A. Bemelman, J.E. van Hooft, and P.J. Tanis have no conflicts of interests or financial ties to disclose for this specific study.

Outside of the submitted work, J.E. van Hooft received a grant from Cook Medicals and a consultancy fee from Boston Scientific and Medtronics.

## AUTHOR CONTRIBUTIONS

All authors contributed to the design, writing, and revision of this manuscript. JV and KB performed the literature search and wrote the initial draft. JV, KB, EC, FB, HW, WB, JH, and PT interpreted the results of the literature search.

## FUNDING INFORMATION

This research did not receive any specific grant from funding agencies in the public, commercial or not‐for‐profit sectors.

## Data Availability

The data that support the findings of this study are available on request from the corresponding author. The data are not publicly available due to privacy or ethical restrictions.
